# Supplementary material for: Recording of Chronic Diseases and Adverse Obstetric Outcomes during Hospitalizations for a Delivery in the National Swiss Hospital Medical Statistics Dataset between 2012 and 2018: An Observational Cross-Sectional Study
Source: Int J Environ Res Public Health. 2022 Jun 28;19(13):7922. doi: 10.3390/ijerph19137922 (PMC9265755; doi:10.3390/ijerph19137922)
Supplement: Supplementary file 1 [file ijerph-19-07922-s001.zip › ijerph-1756292-supplementary.pdf]

SUPPLEMENTAL MATERIAL

Table S1: Codes used to identify the study population, to define demographics and characteristics, as well as to identify recording of chronic diseases and adverse maternal obstetric outcomes. The ".-" implicates that any symbol can follow the preceding symbols.

	**Coding system**[33–35]	**Code**
**Cohort enrolment**		
Delivery (including stillbirth)	ICD-10-GM	Z37.-!
Stillbirth	ICD-10-GM	Z37.1!, Z37.4!, Z37.7!
Multiple birth	ICD-10-GM	Z37.2!, Z37.3!, Z37.5!, Z37.6!
Age at delivery hospitalization	MS (Minimal-datensatz)	_1_1_V01
Length of delivery hospitalization	MS (Minimal-datensatz)	_0_0_V05_2
Type of admission	MS (Minimal-datensatz)	_1_2_V03 (1 = emergency, 2 = planned)
**Chronic diseases**		
Diabetes mellitus (type 1 or 2)	ICD-10-GM	E10.-, E11.-, E12.-, E13.-, O24.0, O24.1, O24.2, O24.3
Thyroid disease	ICD-10-GM	E00.-, E01.-, E02, E03.-, E04.-, E05.-, E06.-, E07.-

Parathyroid diseases	ICD-10-GM	E20.-, E21.-
Cushing syndrome	ICD-10-GM	E24.0, E26.-, E27.- (exclusive E27.2, E27.3, E27.5)
Polycystic ovary syndrome	ICD-10-GM	E28.2
Polyglandular dysfunction	ICD-10-GM	E31.-
Polyarthritis nodosa	ICD-10-GM	M30.0, M30.1, M30.2
Rheumatoid arthritis	ICD-10-GM	M05.-, M06.-, M07.-, M08.-, M09.-, M10.-, M11.-, M12.-, M13.-, M14.-
Systemic lupus erythematosus	ICD-10-GM	M32.-, M33.-, M34.-, M35.0
Inflammatory bowel disease	ICD-10-GM	K50.-, K51.-
Epilepsy	ICD-10-GM	G40.-, G41.-
Multiple Sclerosis	ICD-10-GM	G35.-
Hypertension	ICD-10-GM	I10.-, I11.-, I12.-, I13.-, I15.-
Ischemic heart diseases	ICD-10-GM	I20.-, I21.-, I22.-, I23.-, I24.-, I25.-
Non-ischemic chronic heart diseases	ICD-10-GM	I27.-, I31.-, I34.-, I35.-, I36.-, I37.-, I39.-, I42.-, I43.-, I44.-, I45.-, I46.-, I47.-, I48.-, I49.-, I50.-, I51.-, I52.-
Cerebrovascular diseases	ICD-10-GM	I60.-, I61.-, I62.-, I63.-, I64, I65.-, I66.-, I67.-, I68.-, I69.-
Atherosclerosis	ICD-10-GM	I70.-
Coagulation disorders	ICD-10-GM	D66, D67, D68.-
Chronic lung disease, including asthma	ICD-10-GM	J42, J43, J44.-, J45.-, J47, J60, J61, J62.-, J63.-, J64, J65, J66.-, J67.-, J70.1, J70.3, J70.4, J70.8, J70.9, J84.0 J84.8 J84.9

Human Immunodeficiency Virus (HIV)	ICD-10-GM	B20, B21, B22, B23.-, B24
Mood disorders	ICD-10-GM	F31.-, F33.-, F34.1, F06.32, F38.08, F39.9
Schizophrenia and other paranoid psychoses	ICD-10-GM	F20.-, F21, F22.-, F24, F25.-, F28, F29
Anxiety and personality disorders	ICD-10-GM	F40.-, F60.-, F84.0, F84.1, F84.5, F84.8, F84.9
**Obstetric outcomes**		
Non-instrumental vaginal delivery	CHOP	73.59 (recording was not mandatory in 2012)
Any instrumental vaginal delivery	CHOP	72.-
Forceps delivery	CHOP	72.0.-, 72.1.-, 72.2.-, 72.3.-, 72.4.-, 72.5.-, 72.6.-
Vacuum delivery	CHOP	72.7.-
Any caesarean section	CHOP	74.- (exclusive 74.3, 74.91)
Primary caesarean section	CHOP (since 2014)	74.0X.10, 74.1X.10, 74.2X.10, 74.4X.10, 74.99.10
Secondary caesarean section	CHOP (since 2014)	74.0X.20, 74.1X.20, 74.2X.20, 74.4X.20, 74.99.20
Preterm delivery (<37 completed weeks)	ICD-10-GM	O09.0!, O09.1!, O09.2!, O09.3!, O09.4!, O09.5!
Preterm delivery <25 completed weeks (extremely preterm) [a]	ICD-10-GM	O09.0!, O09.1!, O09.2!, O09.3!
Preterm delivery 26-33 completed weeks [a]	ICD-10-GM	O09.4!

Preterm delivery 34-36 completed weeks [a]	ICD-10-GM	O09.5!
Term delivery (37-41 completed weeks)	ICD-10-GM	O09.6!
Postterm delivery (>41 completed weeks)	ICD-10-GM	O09.7!
Pre-eclampsia / HELLP-syndrome	ICD-10-GM	O11, O14.-
Placental abruption / ischemic placental disease<br>(Abruptio placentae)	ICD-10-GM	O45.0, O45.8, O45.9
Postpartum hemmorhage	ICD-10-GM	O72.0, O72.1, O72.2, O72.3
Gestational diabetes	ICD-10-GM	O24.4, O24.9
Gestational hypertension	ICD-10-GM	O13
Coagulopathy	ICD-10-GM	O45.0, O46.0, O67.0, O72.3
Sepsis	ICD-10-GM	A02.1, A20.7, A22.7, A26.7, A32.7, A39.2, A39.3, A39.4, A40.-, A41.-, A42.7, B00.7, B37.7, B44.7, O75.3, O85, O88.3, R57.2, T88.0
Shock	ICD-10-GM	A48.3, O29.3, O74.4, O75.0, O75.1, R45.7, R57.-, T78.0, T78.2, T79.4, T80.5, T81.1, T88.2
Status asthmaticus	ICD-10-GM	J45.-, J46
Status epilepticus	ICD-10-GM	G40.-, G41.0, G41.1, G41.2, G41.8, G41.9
Acute heart failure	ICD-10-GM	I11.00, I11.01, O29.1, O74.2, O75.4, O89.1
Acute renal failure	ICD-10-GM	I12.00, I12.01, N17.-, O90.4, N99.0

Acute liver failure	ICD-10-GM	K70.40, K72.0, O26.60, O26.68
Acute myocardial infarction	ICD-10-GM	I21.0, I21.1, I21.2, I21.3, I21.4, I21.9
Acute respiratory distress syndrome / respiratory failure	ICD-10-GM	J80.01, J80.02, J80.03, J80.09, J95.2, J96.00, J96.01, J96.09, J98.1, O29.0, O74.1, R09.2
Coma	ICD-10-GM	B19.0, E03.5, E10.01, E11.01, E14.01, E15, K72.7.-, R40.2
Delirium	ICD-10-GM	F05.0, F05.1, F05.8, F05.9, F10.4, F11.4, F12.4, F13.4, F14.4, F15.4, F16.4, F17.4, F18.4, F19.4
Puerperal cerebrovascular disorders	ICD-10-GM	O22.5, O29.2, O74.3, O87.3, O89.2, I60.-, I61.-, I62.-, I63.-, I64.-, I65.-, I66.-, I67.-, I68.-
Pulmonary edema	ICD-10-GM	J81
Pulmonary embolism	ICD-10-GM	I26.0, I26.9, O88.-
Maternal mortality	ICD-10-GM	O89.8, O95, O96.0, O96.1, O96.9, O97.0, O97.1, O97.9
Stay in Intensive Care Unit (ICU)	MS (Minimal-datensatz)	_1_3_V03

[a] Subgroups according to the WHO classification of prematurity was not possible due to pre-defined categories in the ICD-10-GM classification system; and health records of the live-born infant including information on the exact gestational age were not available.

Table S2: Numbers of flow chart of cohort enrolment by year.

	**2012-2018**	**2012**	**2013**	**2014**	**2015**	**2016**	**2017**	**2018**
Number of inpatient stays in the MS datasets	9’965’671	1'353’521	1’374’454	1’401’014	1’430’201	1’467’947	1’470’259	1’468’275
Inclusion criteria: Females	5’332’472	728’616	739’209	752’077	766’395	784’836	781’592	779’747
Inclusion criteria: Delivery during inpatient stay	591’097	81’149	81’850	83’921	85’305	86’713	85’889	86’270
Exclusion of stillbirths: Live-births	588’382	80’783	81’431	83’547	84’911	86’326	85’494	85’890
Exclusion of multiples: Singleton live-births	577‘220	79‘184	79’945	81‘995	83’306	84‘619	83‘902	84’269

Table S3: Demographics and characteristics of the study population (all variables by maternal age category).

	Age	2012-2018	2012	2013	2014	2015	2016	2017	2018
All	All	577220 (100.00)	79184 (100.00)	79945 (100.00)	81995 (100.00)	83306 (100.00)	84619 (100.00)	83902 (100.00)	84269 (100.00)
Age at delivery hospitalization [years]									
<25		51437 (8.91)	8230 (10.39)	8048 (10.07)	7540 (9.20)	7430 (8.92)	7288 (8.61)	6624 (7.90)	6277 (7.45)
25-34		367468 (63.66)	50308 (63.53)	50800 (63.54)	52333 (63.83)	53208 (63.87)	53910 (63.71)	53426 (63.68)	53483 (63.47)
≥35		158315 (27.43)	20646 (26.07)	21097 (26.39)	22122 (26.98)	22668 (27.21)	23421 (27.68)	23852 (28.43)	24509 (29.08)
Length of delivery hospitalization [days], mean (SD) [missing: 31]	All	4.329 (2.87)	4.567 (2.89)	4.467 (2.64)	4.392 (3.41)	4.329 (2.81)	4.261 (2.74)	4.179 (2.77)	4.134 (2.75)
[missing: 1]	<25	4.206 (2.40)	4.358 (2.28)	4.304 (2.49)	4.224 (2.38)	4.170 (2.12)	4.141 (2.28)	4.141 (2.83)	4.051 (2.41)
[missing: 19]	25-34	4.241 (2.76)	4.477 (2.74)	4.376 (2.45)	4.304 (3.59)	4.236 (2.59)	4.180 (2.63)	4.095 (2.60)	4.042 (2.54)

[missing: 11]	≥35	4.574 (3.23)	4.869 (3.39)	4.749 (3.10)	4.656 (3.26)	4.597 (3.43)	4.486 (3.06)	4.379 (3.10)	4.355 (3.21)
Type of hospital admission									
Emergency	All	255927 (44.34)	32090 (40.53)	32828 (41.06)	35526 (43.33)	38658 (46.41)	38562 (45.57)	38571 (45.97)	39692 (47.10)
	<25	25387 (49.36)	3880 (47.15)	3744 (46.52)	3676 (48.75)	3792 (51.04)	3664 (50.27)	3385 (51.10)	3246 (51.71)
	25-34	165023 (44.91)	20505 (40.76)	21040 (41.42)	23047 (44.04)	25060 (47.10)	24773 (45.95)	24923 (46.65)	25675 (48.01)
	≥35	65517 (41.38)	7705 (37.32)	8044 (38.13)	8803 (39.79)	9806 (43.26)	10125 (43.23)	10263 (43.03)	10771 (43.95)
Planned	All	317449 (55.00)	46678 (58.945)	46620 (58.32)	45942 (56.03)	44138 (52.98)	45443 (53.73)	44741 (53.33)	43887 (52.08)
	<25	25703 (49.97)	4304 (52.30)	4255 (52.87)	3814 (50.58)	3585 (48.25)	3572 (49.01)	3185 (48.08)	2988 (47.60)
	25-34	200059 (54.44)	29550 (58.74)	29442 (57.96)	28954 (55.33)	27838 (52.32)	28745 (53.32)	28146 (52.68)	27384 (51.20)
	≥35	91687 (57.91)	12824 (62.11)	12923 (61.26)	13174 (59.55)	12715 (56.09)	13126 (56.04)	13410 (56.22)	13515 (55.14)
Other	All	3844 (0.67)	416 (0.53)	497 (0.62)	527 (0.64)	510 (0.61)	614 (0.73)	590 (0.70)	690 (0.82)

	<25	347 (0.68)	46 (0.56)	49 (0.61)	50 (0.66)	53 (0.71)	52 (0.71)	54 (0.82)	43 (0.69)
	25-34	2386 (0.65)	253 (0.50)	318 (0.63)	332 (0.63)	310 (0.58)	392 (0.73)	357 (0.67)	424 (0.79)
	≥35	1111 (0.70)	117 (0.57)	130 (0.62)	145 (0.66)	147 (0.65)	170 (0.73)	179 (0.75)	223 (0.91)

Abbreviations:

SD = standard deviation

Table S4: Prevalence of chronic diseases recorded during the delivery hospitalization by year between 2012 and 2018.

≥ 1 recorded chronic disease[a]	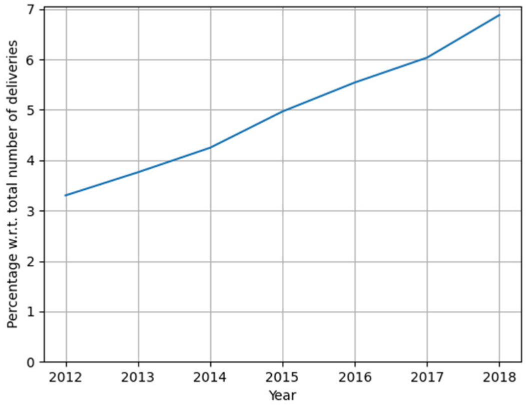
Diabetes mellitus (type 1 or 2)	

<table>
<tr><td></td><td>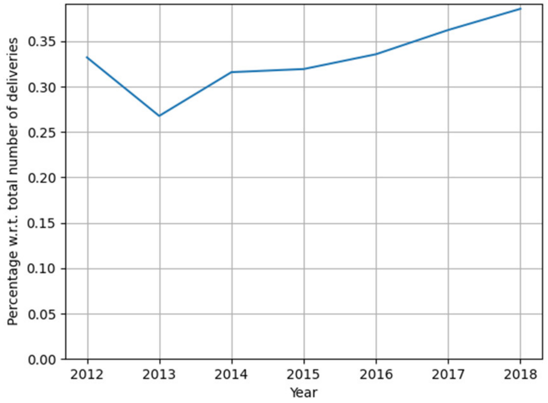
</td></tr>
<tr><td>Thyroid

disorders</td><td>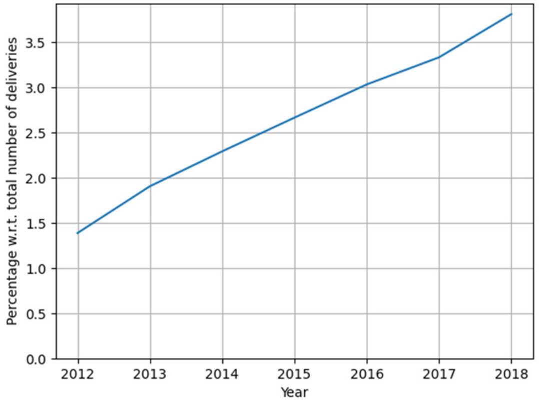
</td></tr>
<tr><td>Epilepsy</td><td></td></tr>
</table>

<table>
<tr>
<td></td>
<td>
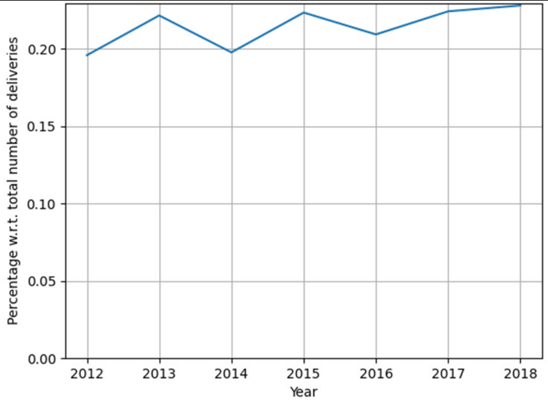

</td>
</tr>
<tr>
<td>Hypertension</td>
<td>
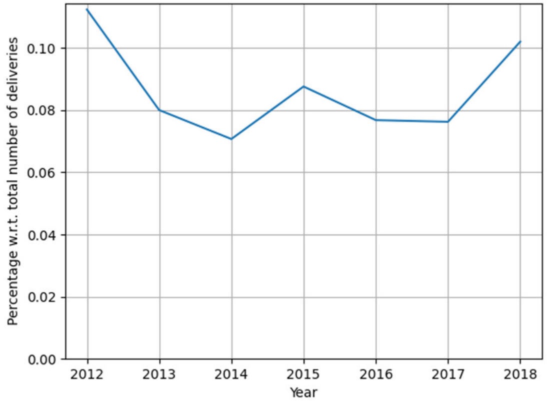

</td>
</tr>
</table>

Chronic lung disease (incl. asthma)	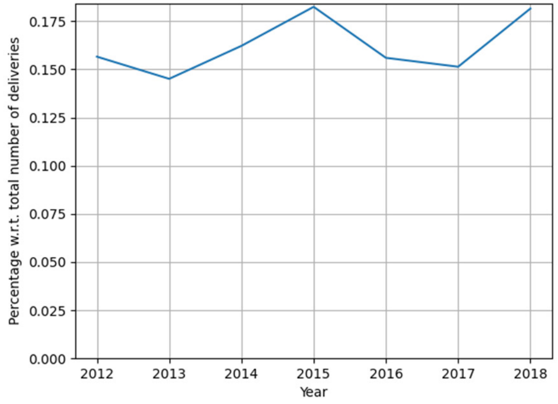

[a] ≥1 recorded chronic disease of the following 23 chronic diseases: diabetes mellitus (type 1 or 2), thyroid disorders, parathyroid disorders, Cushing syndrome, polycystic ovary syndrome (PCOS), polyglandular dysfunction, polyarthritis nodosa, rheumatoid arthritis, systemic lupus erythematosus, inflammatory bowel disease, epilepsy, multiple sclerosis, hypertension, ischemic heart diseases, non-ischemic chronic heart diseases, cerebrovascular diseases, atherosclerosis, coagulation disorders, chronic lung disease, including asthma, human immunodeficiency virus (HIV), mood disorders, schizophrenia/other paranoid psychoses, and anxiety and personality disorders

Table S5: Prevalence of mode delivery, length of gestation, and recorded other adverse maternal obstetric outcomes during the delivery hospitalization in the MS dataset (all variables by maternal age category).

	Age	2012-2018	2012	2013	2014	2015	2016	2017	2018
All	All	577220 (100.00)	79184 (100.00)	79945 (100.00)	81995 (100.00)	83306 (100.00)	84619 (100.00)	83902 (100.00)	84269 (100.00)
	<25	51437 (8.91)	8230 (10.39)	8048 (10.07)	7540 (9.20)	7430 (8.92)	7288 (8.61)	6624 (7.90)	6277 (7.45)
	25-34	367468 (63.66)	50308 (63.53)	50800 (63.54)	52333 (63.83)	53208 (63.87)	53910 (63.71)	53426 (63.68)	53483 (63.47)
	≥35	158315 (27.43)	20646 (26.07)	21097 (26.39)	22122 (26.98)	22668 (27.21)	23421 (27.68)	23852 (28.43)	24509 (29.08)
**Mode of delivery**									
Non-instrumental vaginal delivery[a]	All	321572 (55.71)[a]	39857 (50.34)[a]	44713 (55.93)	45784 (55.84)	46961 (56.37)	47930 (56.64)	47999 (57.21)	48328 (57.35)
	<25	31949 (62.11)[a]	4679 (56.85)[a]	5020 (62.38)	4707 (62.43)	4664 (62.77)	4650 (63.80)	4208 (63.53)	4021 (64.06)
	25-34	210797 (57.37)[a]	25923 (51.53)[a]	29220 (57.52)	30106 (57.53)	30885 (58.05)	31428 (58.30)	31544 (59.04)	31691 (59.25)
	≥35	78826 (49.79)[a]	9255 (44.83)[a]	10473 (49.64)	10971 (49.59)	11412 (50.34)	11852 (50.60)	12247 (51.35)	12616 (51.48)
Instrumental vaginal delivery									
Any instrumental vaginal delivery	All	65991 (11.43)	9266 (11.70)	9132 (11.42)	9307 (11.35)	9463 (11.36)	9560 (11.30)	9620 (11.47)	9643 (11.44)
	<25	6693 (13.01)	1155 (14.03)	1022 (12.70)	941 (12.48)	960 (12.92)	929 (12.75)	871 (13.15)	815 (12.98)

	25-34	44157 (12.02)	6098 (12.12)	6150 (12.11)	6275 (11.99)	6378 (11.99)	6407 (11.89)	6427 (12.03)	6422 (12.01)
	≥35	15141 (9.56)	2013 (9.75)	1960 (9.29)	2091 (9.45)	2125 (9.37)	2224 (9.50)	2322 (9.74)	2406 (9.82)
Forceps delivery	All	6546 (1.13)	1021 (1.29)	995 (1.25)	944 (1.15)	902 (1.08)	965 (1.14)	817 (0.97)	902 (1.07)
	<25	660 (1.28)	135 (1.64)	100 (1.24)	103 (1.37)	96 (1.29)	87 (1.19)	74 (1.12)	65 (1.04)
	25-34	4349 (1.18)	670 (1.33)	683 (1.34)	608 (1.16)	585 (1.10)	642 (1.19)	556 (1.04)	605 (1.13)
	≥35	1537 (0.97)	216 (1.05)	212 (1.01)	233 (1.05)	221 (0.98)	236 (1.01)	187 (0.78)	232 (0.95)
Vacuum delivery	All	59579 (10.32)	8280 (10.46)	8159 (10.21)	8367 (10.20)	8594 (10.32)	8611 (10.18)	8820 (10.51)	8748 (10.38)
	<25	6044 (11.75)	1019 (12.38)	923 (11.47)	836 (11.09)	866 (11.66)	851 (11.68)	800 (12.08)	749 (11.93)
	25-34	39885 (10.85)	5455 (10.84)	5480 (10.78)	5665 (10.83)	5815 (10.93)	5765 (10.69)	5881 (11.01)	5824 (10.89)
	≥35	13650 (8.62)	1806 (8.75)	1756 (8.32)	1866 (8.44)	1913 (8.44)	1995 (8.52)	2139 (8.97)	2175 (8.87)
Cesarean section									
Any cesarean section[b]	All	185680 (32.17)	25662 (32.41)	25977 (32.49)	26953 (32.87)	27035 (32.45)	27319 (32.29)	26403 (31.47)	26331 (31.25)
	<25	12470 (24.24)	1965 (23.88)	2008 (24.95)	1901 (25.21)	1840 (24.76)	1745 (23.94)	1559 (23.54)	1452 (23.13)
	25-34	109881 (29.90)	15358 (30.53)	15395 (30.31)	15994 (30.56)	16032 (30.13)	16175 (30.00)	15534 (29.08)	15393 (28.78)
	≥35	63329 (40.00)	8339 (40.39)	8574 (40.64)	9058 (40.95)	9163 (40.42)	9399 (40.13)	9310 (39.03)	9486 (38.70)

Primary cesarean section	All	NA	NA	NA	7864 (15.03)	7912 (14.87)	7947 (14.74)	7592 (14.21)	7311 (13.67)
	<25	NA	NA	NA	696 (9.23)	718 (9.66)	737 (10.11)	566 (8.55)	545 (8.68)
	25-34	NA	NA	NA	7864 (15.03)	7912 (14.87)	7947 (14.74)	7592 (14.21)	7311 (13.67)
	≥35	NA	NA	NA	5272 (23.83)	5353 (23.62)	5506 (23.51)	5364 (22.49)	5355 (21.85)
Secondary cesarean section	All	NA	NA	NA	12592 (15.36)	12762 (15.32)	12872 (15.21)	12673 (15.11)	12861 (15.26)
	<25	NA	NA	NA	1162 (15.41)	1096 (14.75)	1001 (13.74)	973 (14.69)	890 (14.18)
	25-34	NA	NA	NA	7817 (14.94)	7947 (14.94)	8065 (14.96)	7809 (14.62)	7932 (14.83)
	≥35	NA	NA	NA	3613 (16.33)	3719 (16.41)	3806 (16.25)	3891 (16.31)	4039 (16.48)
**Length of gestation**[c]									
Preterm delivery (<37 completed weeks)	All	23724 (4.11)	3607 (4.56)	3497 (4.37)	3367 (4.12)	3335 (4.00)	3326 (3.93)	3271 (3.90)	3321 (3.94)
	<25	1975 (3.84)	331 (4.02)	333 (4.14)	296 (3.93)	281 (3.78)	258 (3.54)	244 (3.68)	232 (3.70)
	25-34	14444 (3.93)	2200 (4.37)	2158 (4.25)	2049 (3.92)	2021 (3.80)	2045 (3.79)	1983 (3.71)	1988 (3.72)
	≥35	7305 (4.61)	1076 (5.21)	1006 (4.77)	1022 (4.62)	1033 (4.56)	1023 (4.37)	1044 (4.38)	1101 (4.49)
Preterm delivery <25 completed weeks (extremely preterm)	All	1314 (0.23)	167 (0.21)	195 (0.24)	202 (0.25)	184 (0.22)	181 (0.21)	199 (0.24)	186 (0.22)
	<25	137 (0.27)	18 (0.22)	26 (0.32)	30 (0.40)	19 (0.26)	13 (0.18)	19 (0.29)	12 (0.19)

	25-34	769 (0.21)	103 (0.21)	112 (0.22)	112 (0.21)	110 (0.21)	109 (0.20)	117 (0.22)	106 (0.20)
	≥35	408 (0.26)	46 (0.22)	57 (0.27)	60 (0.27)	55 (0.24)	59 (0.25)	63 (0.26)	68 (0.28)
Preterm delivery 26-33 completed weeks	All	5943 (1.03)	895 (1.13)	834 (1.04)	851 (1.04)	877 (1.05)	810 (0.96)	799 (0.95)	877 (1.04)
	<25	517 (1.01)	87 (1.06)	78 (0.97)	89 (1.18)	71 (0.96)	61 (0.84)	65 (0.98)	66 (1.05)
	25-34	3515 (0.96)	515 (1.02)	514 (1.01)	505 (0.97)	507 (0.95)	469 (0.87)	495 (0.93)	510 (0.95)
	≥35	1911 (1.21)	293 (1.42)	242 (1.15)	257 (1.16)	299 (1.32)	280 (1.20)	239 (1.00)	301 (1.23)
Preterm delivery 34-36 completed weeks	All	16478 (2.86)	2548 (3.22)	2469 (3.09)	2315 (2.82)	2275 (2.73)	2336 (2.76)	2275 (2.71)	2260 (2.68)
	<25	1322 (2.57)	226 (2.75)	230 (2.86)	177 (2.35)	191 (2.57)	184 (2.53)	160 (2.42)	154 (2.45)
	25-34	10165 (2.77)	1583 (3.15)	1532 (3.02)	1433 (2.74)	1405 (2.64)	1467 (2.72)	1373 (2.57)	1372 (2.57)
	≥35	4991 (3.15)	739 (3.58)	707 (3.35)	705 (3.19)	679 (3.00)	685 (2.93)	742 (3.11)	734 (3.00)
Term delivery (37-41 completed weeks)	All	493106 (85.43)	69111 (87.28)	68256 (85.38)	69897 (85.25)	70769 (84.95)	71613 (84.63)	71629 (85.37)	71831 (85.24)
	<25	43122 (83.84)	7121 (86.53)	6741 (83.76)	6297 (83.52)	6115 (82.30)	6062 (83.18)	5543 (83.68)	5243 (83.53)
	25-34	313373 (85.28)	43885 (87.23)	43312 (85.26)	44534 (85.10)	45192 (84.94)	45493 (84.39)	45476 (85.12)	45481 (85.04)
	≥35	136611 (86.29)	18105 (87.69)	18203 (86.28)	19066 (86.19)	19462 (85.86)	20058 (85.64)	20610 (86.41)	21107 (86.12)
Postterm delivery (>41 completed weeks)	All	58856 (10.20)	6328 (7.99)	8085 (10.11)	8668 (10.57)	8901 (10.69)	8855 (10.47)	8954 (10.67)	9065 (10.76)

	<25	6192 (12.04)	765 (9.30)	962 (11.95)	944 (12.52)	1003 (13.50)	890 (12.21)	832 (12.56)	796 (12.68)
	25-34	38746 (10.54)	4133 (8.22)	5268 (10.37)	5712 (10.92)	5827 (10.95)	5888 (10.92)	5937 (11.11)	5981 (11.18)
	≥35	13918 (8.79)	1430 (6.93)	1855 (8.79)	2012 (9.10)	2071 (9.14)	2077 (8.87)	2185 (9.16)	2288 (9.34)
**Other adverse maternal obstetric outcomes**									
≥1 recorded other adverse maternal obstetric outcome	All	100501 (17.41)	11752 (14.84)	12468 (15.60)	13219 (16.12)	14299 (17.16)	15163 (17.92)	16281 (19.41)	17319 (20.55)
	<25	8445 (16.42)	1162 (14.12)	1190 (14.79)	1180 (15.65)	1200 (16.15)	1210 (16.60)	1262 (19.05)	1241 (19.77)
	25-34	62303 (16.96)	7268 (14.45)	7870 (15.49)	8122 (15.52)	8938 (16.80)	9476 (17.58)	10026 (18.77)	10603 (19.83)
	≥35	29753 (18.79)	3322 (16.09)	3408 (16.15)	3917 (17.71)	4161 (18.36)	4477 (19.12)	4993 (20.93)	5475 (22.34)
Pre-eclampsia / HELLP-syndrome	All	10355 (1.79)	1183 (1.49)	1444 (1.81)	1426 (1.74)	1457 (1.75)	1571 (1.86)	1628 (1.94)	1646 (1.95)
	<25	935 (1.82)	128 (1.56)	144 (1.79)	143 (1.90)	150 (2.02)	117 (1.61)	131 (1.98)	122 (1.94)
	25-34	6261 (1.70)	724 (1.44)	878 (1.73)	856 (1.64)	860 (1.62)	972 (1.80)	983 (1.84)	988 (1.85)
	≥35	3159 (2.00)	331 (1.60)	422 (2.00)	427 (1.93)	447 (1.97)	482 (2.06)	514 (2.16)	536 (2.19)
Placental abruption / ischemic placental disease (Abruptio placentae)	All	3227 (0.56)	369 (0.47)	416 (0.52)	446 (0.54)	450 (0.54)	516 (0.61)	516 (0.62)	514 (0.61)
	<25	240 (0.47)	33 (0.40)	34 (0.42)	38 (0.50)	30 (0.40)	39 (0.54)	35 (0.53)	31 (0.49)
	25-34	1988 (0.54)	215 (0.43)	254 (0.50)	274 (0.52)	287 (0.54)	306 (0.57)	323 (0.61)	329 (0.62)
	≥35	999 (0.63)	121 (0.59)	128 (0.61)	134 (0.61)	133 (0.59)	171 (0.73)	158 (0.66)	154 (0.63)

Postpartum hemmorhage	All	45899 (7.95)	5457 (6.89)	5671 (7.09)	5996 (7.31)	6506 (7.81)	6965 (8.23)	7479 (8.91)	7825 (9.29)
	<25	4224 (8.21)	574 (6.97)	607 (7.54)	583 (7.73)	615 (8.28)	608 (8.34)	627 (9.47)	610 (9.72)
	25-34	29742 (8.09)	3482 (6.92)	3706 (7.30)	3864 (7.38)	4283 (8.05)	4559 (8.46)	4849 (9.08)	4999 (9.35)
	≥35	11933 (7.54)	1401 (6.79)	1358 (6.44)	1549 (7.00)	1608 (7.09)	1798 (7.68)	2003 (8.40)	2216 (9.04)
Gestational diabetes	All	37990 (6.58)	3932 (4.97)	4349 (5.44)	4781 (5.83)	5435 (6.52)	5854 (6.92)	6451 (7.69)	7188 (8.53)
	<25	2587 (5.03)	309 (3.76)	313 (3.89)	330 (4.38)	380 (5.11)	411 (5.64)	415 (6.27)	429 (6.83)
	25-34	22255 (6.06)	2345 (4.66)	2630 (5.18)	2770 (5.29)	3190 (6.00)	3423 (6.35)	3708 (6.94)	4189 (7.83)
	≥35	13148 (8.31)	1278 (6.19)	1406 (6.66)	1681 (7.60)	1865 (8.23)	2020 (8.63)	2328 (9.76)	2570 (10.49)
Gestational hypertension	All	5889 (1.02)	922 (1.16)	759 (0.95)	857 (1.05)	831 (1.00)	839 (0.99)	818 (0.98)	863 (1.02)
	<25	411 (0.80)	84 (1.02)	71 (0.88)	69 (0.92)	37 (0.50)	53 (0.73)	52 (0.79)	45 (0.72)
	25-34	3524 (0.96)	544 (1.08)	462 (0.91)	497 (0.95)	506 (0.95)	528 (0.98)	474 (0.89)	513 (0.96)
	≥35	1954 (1.23)	294 (1.42)	226 (1.07)	291 (1.32)	288 (1.27)	258 (1.10)	292 (1.22)	305 (1.24)
Coagulopathy	All	1702 (0.30)	106 (0.13)	80 (0.10)	106 (0.13)	136 (0.16)	289 (0.34)	459 (0.55)	526 (0.62)
	<25	120 (0.23)	7 (0.09)	NR	10 (0.13)	9 (0.12)	28 (0.38)	34 (0.51)	28 (0.45)
	25-34	1045 (0.28)	60 (0.12)	52 (0.10)	68 (0.13)	94 (0.18)	171 (0.32)	274 (0.51)	326 (0.61)
	≥35	537 (0.34)	39 (0.19)	24 (0.11)	28 (0.13)	33 (0.15)	90 (0.38)	151 (0.63)	172 (0.70)

Sepsis	All	1722 (0.30)	210 (0.27)	202 (0.25)	231 (0.28)	243 (0.29)	258 (0.31)	309 (0.37)	269 (0.32)
	<25	255 (0.50)	44 (0.54)	33 (0.41)	33 (0.44)	29 (0.39)	35 (0.48)	51 (0.77)	30 (0.48)
	25-34	1033 (0.28)	119 (0.24)	117 (0.20)	128 (0.25)	156 (0.29)	154 (0.29)	190 (0.36)	169 (0.32)
	≥35	434 (0.27)	47 (0.23)	52 (0.25)	70 (0.32)	58 (0.26)	69 (0.30)	68 (0.29)	70 (0.29)
Shock	All	1841 (0.32)	229 (0.29)	304 (0.38)	241 (0.29)	218 (0.26)	275 (0.33)	260 (0.31)	314 (0.37)
	<25	188 (0.37)	27 (0.33)	29 (0.36)	29 (0.39)	27 (0.36)	24 (0.33)	20 (0.30)	32 (0.51)
	25-34	1160 (0.32)	143 (0.28)	211 (0.42)	141 (0.27)	136 (0.26)	176 (0.33)	159 (0.30)	194 (0.36)
	≥35	493 (0.31)	59 (0.29)	64 (0.30)	71 (0.32)	55 (0.24)	75 (0.32)	81 (0.34)	88 (0.36)
Status asthmaticus	All	914 (0.16)	119 (0.15)	114 (0.14)	128 (0.16)	150 (0.18)	130 (0.15)	121 (0.14)	152 (0.18)
	<25	78 (0.15)	9 (0.11)	14 (0.17)	13 (0.17)	13 (0.18)	11 (0.15)	8 (0.12)	10 (0.16)
	25-34	559 (0.15)	82 (0.16	72 (0.142)	77 (0.15)	88 (0.17)	80 (0.15)	77 (0.14)	83 (0.16)
	≥35	277 (0.18)	28 (0.14)	28 (0.13)	38 (0.17)	49 (0.22)	39 (0.17)	36 (0.15)	59 (0.24)
Status epilepticus	All	1237 (0.21)	155 (0.20)	177 (0.22)	162 (0.20)	186 (0.22)	177 (0.21)	188 (0.22)	192 (0.23)
	<25	116 (0.23)	18 (0.22)	19 (0.24)	17 (0.23)	15 (0.20)	11 (0.15)	19 (0.29)	17 (0.27)
	25-34	807 (0.22)	107 (0.21)	122 (0.24)	106 (0.20)	121 (0.23)	115 (0.21)	106 (0.20)	130 (0.24)
	≥35	314 (0.20)	30 (0.15)	36 (0.17)	39 (0.18)	50 (0.22)	51 (0.22)	63 (0.26)	45 (0.18)
Acute heart failure	All	276 (0.05)	34 (0.04)	33 (0.04)	34 (0.04)	30 (0.04)	34 (0.04)	52 (0.06)	59 (0.07)
	<25	-	-	-	-	-	-	-	-
	25-34	-	-	-	-	-	-	-	-
	≥35	-	-	-	-	-	-	-	-
Acute renal failure	All	138 (0.02)	17 (0.02)	14 (0.02)	17 (0.02)	14 (0.02)	8 (0.01)	29 (0.04)	39 (0.05)

	<25	-	-	-	-	-	-	-	-
	25-34	-	-	-	-	-	-	-	-
	≥35	-	-	-	-	-	-	-	-
Acute liver failure	All	9 (0.00)	NR	NR	NR	NR	NR	NR	NR
	<25	-	-	-	-	-	-	-	-
	25-34	-	-	-	-	-	-	-	-
	≥35	-	-	-	-	-	-	-	-
Acute myocardial infarction	All	8 (0.00)	NR	NR	NR	NR	NR	NR	NR
	<25	-	-	-	-	-	-	-	-
	25-34	-	-	-	-	-	-	-	-
	≥35	-	-	-	-	-	-	-	-
Acute respiratory distress syndrome / respiratory failure	All	150 (0.03	10 (0.01)	25 (0.03)	15 (0.02)	24 (0.03)	27 (0.03)	29 (0.04)	20 (0.04)
	<25	-	-	-	-	-	-	-	-
	25-34	-	-	-	-	-	-	-	-
	≥35	-	-	-	-	-	-	-	-
Coma	All	15 (0.00)	NR	NR	NR	NR	NR	NR	NR
	<25	-	-	-	-	-	-	-	-
	25-34	-	-	-	-	-	-	-	-
	≥35	-	-	-	-	-	-	-	-
Delirium	All	12 (0.00)	NR	NR	NR	NR	NR	NR	NR
	<25	-	-	-	-	-	-	-	-
	25-34	-	-	-	-	-	-	-	-

	≥35	-	-	-	-	-	-	-	-
Puerperal cerebrovascular disorders	All	119 (0.02)	18 (0.02)	8 (0.01)	18 (0.02)	12 (0.01)	22 (0.03)	16 (0.02)	25 (0.03)
	<25	-	-	-	-	-	-	-	-
	25-34	-	-	-	-	-	-	-	-
	≥35	-	-	-	-	-	-	-	-
Pulmonary edema	All	108 (0.02)	22 (0.03)	13 (0.02)	10 (0.01)	20 (0.02)	15 (0.02)	13 (0.02)	15 (0.02)
	<25	-	-	-	-	-	-	-	-
	25-34	-	-	-	-	-	-	-	-
	≥35	-	-	-	-	-	-	-	-
Pulmonary embolism	All	144 (0.03)	23 (0.03)	23 (0.03)	26 (0.03)	19 (0.02)	17 (0.02)	10 (0.01)	26 (0.03)
	<25	-	-	-	-	-	-	-	-
	25-34	-	-	-	-	-	-	-	-
	≥35	-	-	-	-	-	-	-	-
Maternal mortality	All	31 (0.01)	NR	5 (0.01)	NR	6 (0.01)	NR	5 (0.01)	9 (0.01)
	<25	-	-	-	-	-	-	-	-
	25-34	-	-	-	-	-	-	-	-
	≥35	-	-	-	-	-	-	-	-
Stay in ICU [missing: 103]	All	3841 (0.67)	383 (0.48)	915 (1.15)	488 (0.60)	562 (0.68)	494 (0.58)	513 (0.61)	486 (0.58)
[missing: 6]	<25	288 (0.56)	29 (0.35)	80 (0.99)	41 (0.54)	33 (0.44)	27 (0.37)	47 (0.71)	31 (0.49)
[missing: 65]	25-34	2366 (0.64)	240 (0.48)	613 (1.21)	286 (0.58)	326 (0.61)	310 (0.58)	294 (0.55)	297 (0.56)
[missing: 32]	≥35	1187 (0.75)	114 (0.55)	222 (1.05)	161 (0.73)	203 (0.90)	157 (0.67)	172 (0.72)	158 (0.45)

Abbreviations:

MS = Hospital Medical Statistics

NA = not available (no CHOP codes available in 2012 and 2013)

NR = not reported (because cell size <5 patients)

HELLP = Hemolysis, Elevated Liver enzymes and Low Platelets

ICU = Intensive Care Unit

[a] Non-instrumental vaginal delivery: The procedure code 74.91 was not mandatory to record in 2012, which affects the numbers in 2012 and 2012-2018

[b] Remaining pregnancies are with other or unknown type of cesarean section than primary or secondary cesarean section

[c] Remaining pregnancies are with unknown length of gestation (either a ICD-10-GM code O09.9! or no ICD-10-GM which indicates the length of gestation)
